# NPT1220-312, a TLR2/TLR9 Small Molecule Antagonist, Inhibits Pro-Inflammatory Signaling, Cytokine Release, and NLRP3 Inflammasome Activation

**DOI:** 10.1155/2022/2337363

**Published:** 2022-02-27

**Authors:** Agata Habas, Srinivasa Reddy Natala, Jon K. Bowden-Verhoek, Emily M. Stocking, Diana L. Price, Wolfgang Wrasidlo, Douglas W. Bonhaus, Martin B. Gill

**Affiliations:** ^1^Arrowhead Pharmaceuticals Inc., 11404 Sorrento Valley Road, San Diego, CA 92121, USA; ^2^Zentalis Pharmaceuticals, 10835, Road to the Cure Suite 205, San Diego, CA 92121, USA; ^3^Neuropore Therapies Inc., 11585 Sorrento Valley Rd. Suite 106, San Diego, CA 92121, USA; ^4^Libra Therapeutics, 3210 Merryfield Row, San Diego, CA 92121, USA

## Abstract

Toll-like receptors (TLRs) play a critical role in innate immune system responses to damage-associated molecular patterns (DAMPs) and pathogen-associated molecular patterns (PAMPs). A growing body of evidence suggests that excessive TLR-mediated innate immune system activation can lead to neuronal damage and precipitate or perpetuate neurodegenerative diseases. Among TLR subtypes, both TLR2 and TLR9 have been implicated in neurodegenerative disorders with increased expression of these receptors in the central nervous system being associated with pro-inflammatory signaling and increased burdens of pathologic aggregated proteins. In the current study, we characterized the actions of a combined TLR2/TLR9 antagonist, NPT1220-312, on pro-inflammatory signaling and cytokine release in monocyte/macrophage-derived heterologous cells, human microglia, and murine and human whole blood. NPT1220-312 potently blocked TLR2- and TLR9-mediated release of inflammatory cytokines in monocyte/macrophage cells and in human microglia. NPT1220-312 also blocked TLR2-mediated activation of the NLR family pyrin domain containing 3 (NLRP3) inflammasome including IL-1*β*, IL-18, and apoptosis-associated speck-like protein containing a CARD (ASC) release to the culture medium of human differentiated macrophages. The ability of NPT1220-312 to inhibit TLR2 mediated pro-inflammatory release of chemokines and cytokines in situ was demonstrated using murine and human whole blood. Together, these findings suggest that blockade of TLR2 and TLR9 may reduce inappropriate production of pro-inflammatory cytokines and chemokines from peripheral and central immune cells and thus potentially provide therapeutic benefit in neuroinflammatory/neurodegenerative disorders.

## 1. Introduction

The innate immune system is the body's first defense mechanism against microbial infection. It relies on host pattern recognition receptors (PRRs) to rapidly recognize and respond to signals coming from the pathogens themselves or injured host cells [1]. This controlled innate inflammatory response is critical for the elimination of pathogens. Conversely, dysregulated immune responses, mediated in part by macrophages in the periphery and microglia in the central nervous system (CNS), can contribute to pathological conditions including autoimmune diseases and neurodegenerative disorders. Thus, pro-inflammatory actions of peripheral macrophages may contribute to the pathology of Guillain–Barré syndrome (GBS), an autoimmune disorder affecting the peripheral nervous system (PNS), while pro-inflammatory signaling arising, in part, from dysregulated microglia contributes to the progression of CNS neurodegenerative disorders such as amyotrophic lateral sclerosis (ALS) and Parkinson's disease (PD) [2–8].

The pathogen or host signals that activate immune responses are mediated by PRRs, which include the family of toll-like receptors (TLRs). TLRs identify and bind pathogen-associated molecular patterns (PAMPs), such as bacterial- and viral-derived carbohydrates, nucleic acids and lipoproteins and also endogenous, host-derived danger, or damage-associated molecular patterns (DAMPs), such as misfolded proteins and mitochondrial DNA, that are released by damaged or dying cells [9–13]. DAMP- or PAMP-mediated activation of TLRs recruits intracellular signaling scaffolds that interact, in part through an intracellular Toll/interleukin-1 receptor (TIR) homology domain, to produce cellular sequelae leading to nuclear factor kappa B (NF*κ*B) mediated transcription and the release of inflammatory cytokines and chemokines [9, 14].

The interplay between DAMPs, and more specifically disease-associated pathologic proteins such as alpha-synuclein, and TLRs is complex. Disease-associated proteins can directly or indirectly activate TLRs with a consequent triggering of immune responses while, in a reciprocal manner, the activation of these PRRs can impair neuronal and non-neuronal processes for the lysosomal degradation of these same disease-associated proteins. This interplay between the immune response and protein clearance mechanisms can result in increased cell-to-cell transmission of pathologic proteins that further contributes to the progression of neurodegenerative disorders [15–18]. One important aspect of TLR-mediated signaling in disease pathology is activation of the NLRP3 (NOD-like receptor protein 3) inflammasome, a multiprotein complex that has been implicated in a wide range of diseases. Inflammasome signaling entails pro-caspase-1-mediated cleavage of pro-IL-1*β* and pro-IL-18, and the subsequent release of the mature pro-inflammatory cytokines via the adaptor molecule ASC (apoptosis-associated speck-like protein containing a CARD) to initiate pyroptosis, an inflammatory form of programmed cell death. NLRP3 signaling requires a two-step process: priming and activation. The priming step can be provided by microbial components or endogenous molecules, which can act via TLRs to increase pro-IL-1*β* and pro-IL-18 expression, while the activation step may be driven by aberrant aggregated host proteins such as *α*-synuclein or amyloid-*β* [19–26]. In this manner, the two-step triggering of the NLRP3 inflammasome, involving priming by DAMPs acting, in part, through TLRs and activation by disease-associated aggregated host proteins provides a point of convergence for pathological processes that dysregulate immune responses and impair the degradation of disease-associated pathologic proteins.

Several subtypes of TLR have been implicated in neurodegenerative disorders including TLR2 and TLR9. Both TLR2 and TLR9 are expressed in microglia; both are involved in NLRP3 inflammasome signaling [20, 22, 27]; and the activation of either receptor can produce neurotoxicity [28–31]. Moreover, both TLR2 and TLR9 are increased in CNS tissue from patients with neurodegenerative disorders and in animal models of neurodegeneration, with disease-relevant beneficial effects achieved in the animal models by genetic or pharmacological inactivation of these receptors [13, 15, 28, 32–39]. These findings suggest that not only are TLR2 and TLR9 upregulated in neurodegenerative disorders and contribute to the progressive pathology, but also that a therapeutic benefit may be achieved by their blockade.

In the studies reported here, we characterized the actions of NPT1220-312, a dual TLR2 and TLR9 small molecule antagonist, on inflammatory signaling in peripheral and central immune cells, on TLR2-mediated priming of the NLRP3 inflammasome, and on TLR2-evoked release of cytokines and chemokines in murine and human whole blood.

## 2. Experimental Procedures

### 2.1. Reagents and Media

NPT1220-312 was synthesized by PepTech Corporation (Bedford, MA, USA). The purity of NPT1200-312 was reported as >99.5%. Synthetic diacylated lipoprotein (Pam_2_CSK_4_, TLR2/6 ligand, cat no. tlrl-pm2s-1), synthetic triacylated lipoprotein (Pam_3_CSK_4_, TLR1/2 ligand, cat. ID: tlrl-pms), polyinosinic-polycytidylic acid (poly I:C, TLR3 ligand, cat. ID: tlrl-pic), lipopolysaccharide (LPS-EK-Ultrapure, TLR4 ligand, cat. ID: tlrl-peklps), 9-benzyl-8-hydroxyadenine derivative (CL-264, TLR7 ligand, cat. ID: tlrl-c264e), resiquimod (R848, TLR7/8 ligand, cat. ID: tlrl-r848), ODN2006 (human TLR9 ligand, cat. ID: tlrl-2006), ODN1826 (murine TLR9 ligand, cat. ID: tlrl-1826), anti-TLR2 antibody (mab2-mtlr2), and MCC950 (NLRP3 inhibitor, cat. ID: inh-mcc) were purchased from InvivoGen (San Diego, CA USA). Recombinant human tumor necrosis factor-*α* (TNF*α*, PHC3011) was purchased from Fisher Scientific. TLR9 inhibitor (E6446) was purchased from Selleckchem (Houston, TX, USA). TLR2 reference antagonists including c29 and O-vanillin were synthesized in-house; TLR1/2 inhibitor, CuCPT22, was purchased from Tocris (cat. ID: HY-108471). All agonists were dissolved using the manufacturer's recommended procedure, vortexed until completely solubilized, and either used immediately or stored in aliquots at −20°C. *Propionibacterium acnes (P. acnes*, 6919, now named *Corynebacterium acnes*) and *Porphyromonas gingivalis* (*P. gingivalis*, 33277) were purchased from ATCC (Manassas, Virginia). For consistency with the previous reporting, we will refer to *C. acnes* by its former name, *P. acnes*. Both bacteria upon resuspension in PBS were heat-inactivated (25 min at 60°C for *P. acnes* or 1 h at 70°C for *P. gingivalis*) and then sonicated for 15 minutes in a water bath sonicator. Bacteria protein concentration was determined using a BCA protein assay kit (23225, Thermo Scientific). Heat-inactivated bacteria were aliquoted and used immediately or stored at −80°C. NPT1220-312 dissolved with sonication in a water batch as a 20 mM stock in 100% DMSO. Serial dilutions were prepared in 100% DMSO and then further diluted in the test medium. The final concentration of DMSO used in the cellular assays was 1% or below.

### 2.2. Cell Culture

Human embryonic kidney (HEK) Blue hTLR2 secreted embryonic alkaline phosphatase (SEAP) reporter 293 cells, expressing the human TLR2 gene; HEK-Blue hTLR3 SEAP reporter 293 cells, expressing the human TLR3 gene; HEK-Blue hTLR4 SEAP reporter 293 cells, expressing the human TLR4 gene; HEK-Blue hTLR7 SEAP reporter 293 cells, expressing the human TLR7 gene; and HEK-Blue hTLR8 SEAP reporter 293 cells, expressing the human TLR8 gene. HEK Blue cells expressing recombinant human TLRs were obtained by cotransfection of the human TLR2, TLR3, TLR4, TLR7, TLR8, or TLR9 and SEAP (secreted embryonic alkaline phosphatase) genes into HEK293 cells. The SEAP reporter gene was placed under the control of a minimal promoter fused to five NFĸB and AP-1 binding sites. Stimulation with TLRs ligands activates NFĸB and AP-1 that induce the production of SEAP. Therefore, these cells are designed for studying the stimulation of TLRs by monitoring the activation of NFĸB/AP-1. These NFĸB reporter cell lines were used as a primary screening tool due to their robust and well-characterized responses to TLRs activators and inhibitors. HEK-Blue hTLR9 SEAP reporter 293 cells, expressing the human TLR9 gene; RAW-Blue cells derived from RAW 264.7 macrophages, stably expressing the NF-*κ*B and AP-1-inducible SEAP reporter gene; and THP1-XBlue cells that stably express NF-*κ*B and the AP-1-inducible SEAP reporter gene were obtained from InvivoGen. THP1-Blue NFĸB cells were derived from a human THP-1 monocyte cell line by stable integration of an NFĸB-inducible SEAP reporter construct. As a result, THP1-Blue NFĸB cells allow the monitoring of NFĸB activation by determining the activity of SEAP. Similarly, RAW-Blue cells are derived from the murine RAW 264.7 macrophages with chromosomal integration of a secreted embryonic alkaline phosphatase (SEAP) reporter construct inducible by NF-*κ*B and AP-1. THP1-Blue NFĸB cells, RAW-Blue cells, and primary human microglia are representative of peripheral and central cell types important in immune responses; they endogenously express TLR2 or/and TLR9 and represent more physiologically relevant cell lines than the HEK cells expressing recombinant receptors and therefore were used as a secondary screening tool. These cells were used to confirm that the inhibitor actions of NPT1220-312 were not restricted to artificial agonists in cell lines expressing recombinant receptors. The growth medium for HEK-Blue hTLR2 and HEK-Blue hTLR4 cells contained: DMEM (Gibco), 1x GlutaMax (Gibco), 10% heat-inactivated fetal bovine serum (Gibco), pen-strep (50 U/mL penicillin, 50 *μ*g/mL streptomycin, Gibco), and 100 *μ*g/mL normocin (InvivoGen). The growth medium was supplemented with antibiotics, 1x HEK-Blue Selection for hTLR2 or hTLR4 cells, 30 *μ*g/ml blasiticidin and 100 *μ*g/ml zeocin for hTLR3, and hTLR8 cells, 10 *μ*g/ml blasticidin and 100 *μ*g/ml zeocin for hTLR7 and hTLR9 cells, and 10 *μ*g/ml blasticidin for murine RAW-Blue cells (InvivoGen). The growth medium for THP1-XBlue cells contained: RPMI 1640 (2 mM L-glutamine, 1.5 g/L sodium bicarbonate, 4.5 g/L glucose, 10 mM HEPES, and 1.0 mM sodium pyruvate, Gibco) with 10% heat-inactivated fetal bovine serum (Gibco), 100 *μ*g/mL normocin (InvivoGen), and pen-strep (50 U/mL penicillin, 50 *μ*g/mL streptomycin, Gibco). The THP1 growth medium was supplemented with 200 *μ*g/mL zeocin (InvivoGen). Immortalized human microglia (cat. ID: P10354-IM) were purchased from Innoprot. Microglial cell medium (cat. ID: P60116) with all components was purchased from Innoprot. Innoprot microglial basal medium was supplemented with 5% HD-fetal bovine serum, 1% microglia growth supplement (MCGS), and 1% penicillin-streptomycin solution. For experiments involving human macrophages, THP1-Blue NF-kB cells (InvivoGen) were differentiated by 6 h incubation with 40.5 nM Phorbol 12-myristate 13-acetate (PMA, tlrl-pma, InvivoGen) followed by 1X wash in prewarmed PBS and cultured in growth medium for 3–4 days. Before the treatment cells were washed one more time with prewarmed PBS and then were incubated in growth medium RPMI 1640 (2 mM∙L-glutamine, 1.5 g/L sodium bicarbonate, 4.5 g/L glucose, 10 mM HEPES, and 1.0 mM sodium pyruvate, Gibco) with 10% heat-inactivated fetal bovine serum (Gibco), 100 *μ*g/mL normocin (InvivoGen), and pen-strep (50 U/mL penicillin, 50 *μ*g/mL streptomycin, Gibco). The growth medium was supplemented with a selective antibiotic, 10 *μ*g/mL blasticidin (InvivoGen).

### 2.3. TLR2 Antagonism Assay and Quantification of Nuclear NF-kB Activity

Fifty microliter per well of test compound or vehicle was added to 96-well plates. Then, 150 *μ*l of cell suspension at 1 × 10 [5] cell per well (HEK-Blue hTLR2, HEK-Blue hTLR3, HEK-Blue hTLR4, HEK-Blue hTLR7, HEK-Blue hTLR8, HEK-Blue hTLR9, and RAW-Blue) or 1.2 × 10 [5] cells per well (THP1-XBlue) were plated. The cells were incubated at 37°C, 5% CO_2_ for 2 h. Next, 50 *μ*l/well of an agonist (Pam_2_CSK_4_, Pam_3_CSK_4_, TNF*α*, Poly (I:C), LPS, CL-264, TL8-506, ODN2006, or ODN1826) was added at concentrations corresponding to three times their respective EC_50_ values, unless described differently in the figure legend, as determined by agonist concentration-response curves. The plates were then incubated at 37°C, 5% CO_2_ for 18 h. Following the 18 h incubation, SEAP activity from cell culture supernatants was quantified. Quanti-Blue medium for detection and quantification of alkaline phosphatase was prepared. Quanti-Blue is provided as 100X-concentrated Quanti Blue (QB) reagents and 100X-concentrated QB buffer (rep-qbs, InvivoGen). One vial (1 ml) of QB buffer and QB reagent (1 ml) were dissolved in 98 mL of endotoxin-free water, warmed to 37°C for 30 minutes, and then filtered on a 0.2 *μ*m membrane. Next, 20 *μ*l supernatant of SEAP-expressing cells was added per well to a 96-well plate, and then 200 *μ*l of Quanti-Blue was added per well. Plates were incubated at room temperature, and SEAP activity was assessed by reading the OD at 655 nm.

### 2.4. Inflammasome Activation

On day one of the experiment, differentiated human macrophages (described above) were pretreated with test compounds or vehicles for 1 h. Human macrophages were primed for 3 h with 1.5 ng/ml Pam_3_CSK_4_ and then activated with 100 *μ*g/ml monosodium urate crystals (MSU, tlrl-msu, InvivoGen) for 18 h. Cell culture media was then removed for quantification of released SEAP, IL-1*β*, IL-18, and ASC.

### 2.5. Mouse Whole Blood Assay

Male wild-type BDF1 mice were utilized for the whole blood assay evaluations under Explora Biosciences animal care and use protocol (ACUP), number E15-016. Animals were housed up to 5 per cage and received free access to food and water. Murine whole blood (WB), collected by Neuropore personnel into 4 ml vials containing 95 USP sodium heparin (368037, BD Biosciences) via cardiac stick, was checked for hemolysis after centrifuging at 2,500 g for 5 min. Only non-hemolyzed WB samples were used for subsequent efforts. Freshly isolated WB was diluted 1:10 in RPMI 1640 medium supplemented with 1% PS and stored in a 37°C water bath until use. NPT1220-312 was preincubated with the diluted whole blood for 2 h (37°C, 5% CO_2_) prior to the addition of an EC_70_ concentration of Pam_3_CSK_4_ (∼1 *μ*g/ml) for 48 h. After 48 h, cell culture supernatant containing cytokines and chemokines elicited by Pam_3_CSK_4_-mediated receptor activation was collected and stored at −80°C until analysis via Luminex MAGPix.

### 2.6. Human Whole Blood Assay

Human whole blood (WB) was collected in 4 mL vials containing sodium heparin (Fisher 23-021-017) in the San Diego Blood Bank and obtained from them in 95 USP sodium heparin (368037, BD Biosciences) and then checked for hemolysis by centrifuging at 2,500 g for 5 min. Only non-hemolyzed WB samples were used for subsequent efforts. Whole blood was diluted 1:10 in RPMI 1640 medium supplemented with 1% PS and stored in a 37°C water bath until use. A dilution series of NPT1220-312 was preincubated with the diluted whole blood for 2 h in a cell culture incubator (37°C, 5% CO_2_). An EC_70_ concentration of Pam_3_CSK_4_ (∼2 ng/mL) was then added to the diluted WB plus compound well and incubated for 72 h in a cell culture incubator. After 72 h, cell culture supernatant containing cytokines and chemokines elicited by Pam_3_CSK_4_-mediated activation was collected and stored at −80°C until analysis via Luminex MAGPix.

### 2.7. Enzyme-Linked Immunosorbent Assay (ELISA)

Human Interleukin-8 (IL-8) concentrations in cell culture supernatants were quantified using a single-analyte ELISA kit according to the manufacturer's instructions (Qiagen). ASC concentrations in cell culture supernatants were quantified using human PYCARD ELISA Kit according to the manufacturer's instructions (cat. ID: OKEH01695, Aviva Systems Biology).

### 2.8. Luminex/Milliplex Assay for CNS Microglia, Inflammasome Activation, and Mouse and Human Whole Blood Assays

Concentrations of human Interleukin-1*β* (IL-1*β*), human Interleukin-18 (IL-18), human Interleukin-6 (IL-6), human Interleukin-8 (IL-8), murine KC, murine macrophage inflammatory protein-2 (MIP-2), and murine Interleukin-6 (IL-6) in cell culture supernatants were quantified using human IL-1*β* or human IL-18 multiplex magnetic bead kits and multiplex kits for murine (KC, IL-6, MIP-2) and multiplex kits for human (IL-6 and IL-8), according to the manufacturer's instructions (Millipore-Sigma). Cell culture or diluted WB supernatant samples were centrifuged (10,000 g for 10 min, 4°C) to pellet any cellular debris before use. The samples were read, and the bead counts were obtained using Luminex MAGPix instrument and xPONENT software. A serial dilution of known standards was used to generate standard curves.

### 2.9. Statistics

GraphPad Prism software was used to obtain EC_50_ and IC_50_ values from concentration-response curves and to perform statistical analysis. IC_50_ values are presented with 95% confidence intervals. When comparing two groups, a two-tailed *t*-test was used in conjunction with a Welsh correction if the group variances were measured as statistically different. For analyses of assays involving three or more groups, a one-way analysis of variance (ANOVA) was conducted with a Dunnett's post hoc analysis to determine statistical significance between defined comparisons referencing a common control value. Statistical significance was defined as a *p*-value of less than or equal to 0.05 for all assays. For antagonist potency (IC_50_) evaluations, data were normalized to a defined control, and a four-parameter non-linear regression fit was conducted with the “top” defined as 100% and the “bottom” defined as 0%, unless stated otherwise. Schild regression and Gaddum plot analysis were conducted as described previously [40]. The figures presented in this report are a representative data set from a single experiment, which was selected from multiple, separate independent biological replications.

## 3. Results

### 3.1. NPT1220-312 Is a Potent Inhibitor of TLR2 and TLR9

**Figure 1:**
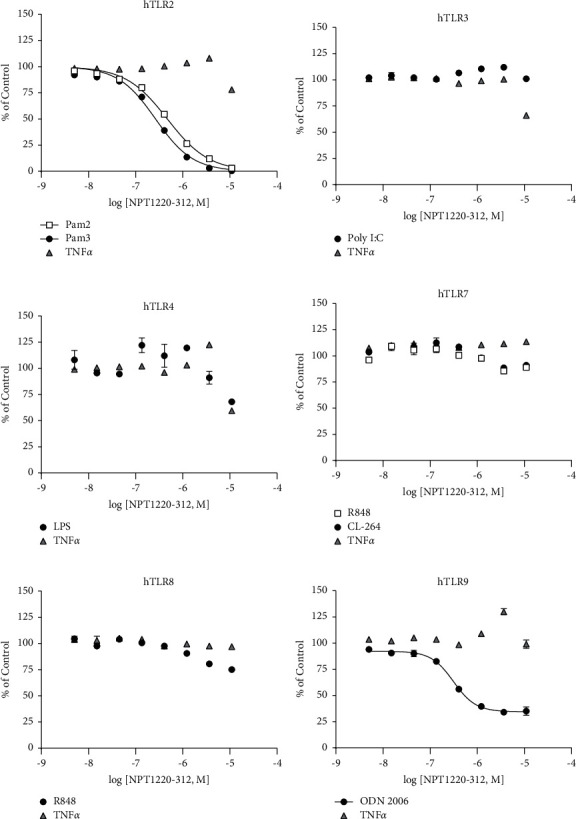
NPT1220-312 selectively inhibits TLR2 and TLR9. NPT1220-312 inhibition of (a) TLR1/2 as activated by Pam_3_CSK_4_ and TLR2/6 as activated by Pam_2_CSK_4_ in HEK293 cells overexpressing hTLR2; (b) TLR3 as activated by Poly I:C in HEK293 cells overexpressing hTLR3; (c) TLR4 as activated by LPS in HEK293 cells overexpressing hTLR4; (d) TLR7 as activated by R848 and as activated by CL-264 in HEK293 cells overexpressing hTLR7; (e) TLR8 as activated by R848 in HEK293 cells overexpressing hTLR8; and (f) TLR9 as activated by ODN2006 in HEK293 cells overexpressing hTLR9. TNF*α* was incorporated as a control for non-specific inhibition of signaling in each assay.

NPT1220-312 was found to be a concentration-dependent inhibitor of TLR1/2 (IC_50_ = 260 nM; 230–300 nM), TLR2/6 (IC_50_ = 470 nM; 430–520 nM), and TLR9 (IC_50_ 317 nM; 260–370 nM with a maximum inhibition of 70% of the evoked response). NPT1220-312 had little or no inhibitory effect on TLR3, TLR4, TLR7, TLR8, or the assay specificity control TNF*α* (Figure 1). NPT1220-312 was found to be approximately 50-fold more potent at TLR2 than other previously disclosed small-molecule TLR2 inhibitors (Table 1).

### 3.2. NPT1220-312 Blocks Activation of TLR2 That Is Mediated by Synthetic and Endogenous Agonists

**Figure 2:**
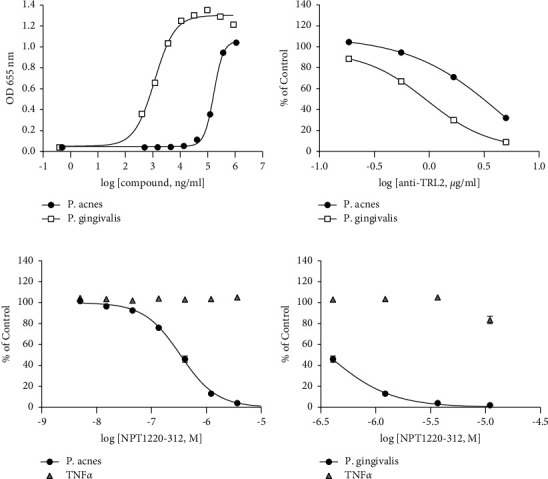
NPT1220-312 blocks TLR2 signaling mediated by natural TLR2 pathogen agonists: (a) concentration-response curves for *P. gingivalis* or *P. acnes* in hTLR2 HEK293 cells, (b) blockade of *P. acnes* or *P. gingivalis* signaling by an anti-TLR2 specific antibody, (c) blockade of *P. acnes*-evoked response by NPT1220-312, and (d) blockade of *P. gingivalis*-evoked response by NPT1220-312 ( ^*∗*^*p* ≤ 0.05).

To further characterize the interaction of NPT1220-312 with TLR2, the ability of NPT1220-312 to inhibit TLR2 activation by both artificial and natural activators of signaling was examined. NPT1220-312 potently inhibited TLR2 activation by *P. acnes* and *P. gingivalis* indicating that the inhibitory actions of NPT1220-312 are not restricted to synthetic TLR2 agonists (Figure 2).

### 3.3. NPT1220-312 Is a Non-Competitive, Allosteric Antagonist of TLR2 and TLR9

**Figure 3:**
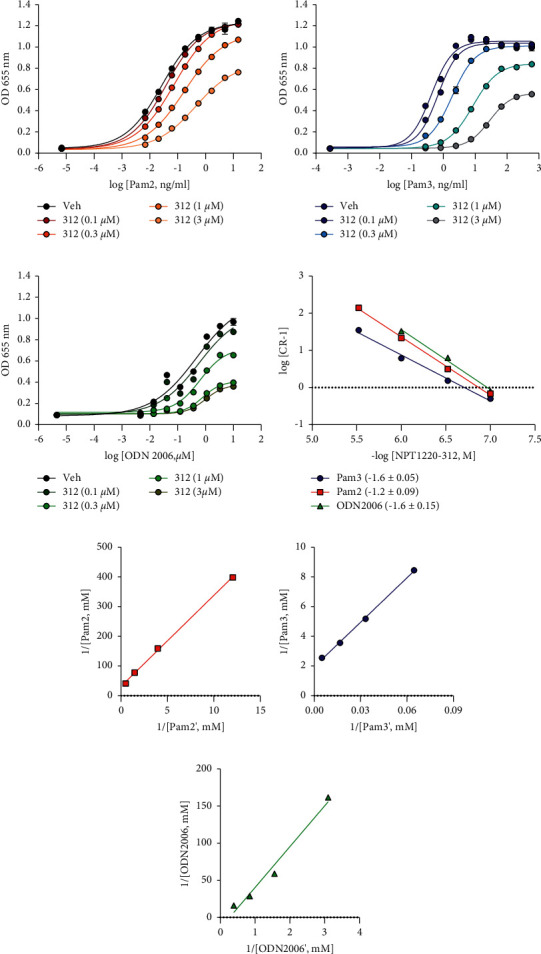
NPT1220-312 is an allosteric antagonist at TLR2 and TLR9. NPT1220-312 suppressed: (a) Pam_2_CSK_4_-, (b) Pam_3_CSK_4_-, and (c) ODN2006-evoked SEAP release (OD655 nm) to the medium in hTLR2 HEK293 cells; (d) the dose-effect curve for an agonist determined in the presence of various concentrations of an antagonist, Schild plots; and (e–g) dose ratios derived from Gaddum analysis of EC_50_ and IC_50_ values for (e) Pam_2_CSK_4_, (f) Pam_3_CSK_4_, and (g) ODN2006. NPT1220-312 concentration dependently suppressed the maximum TLR2 or TLR9 agonist-evoked responses in a manner consistent with a non-competitive interaction [40].

The ability of NPT1220-312 to block multiple distinct TLR2 agonists, together with molecular modeling studies of the putative binding site in the toll/interleukin-1 receptor (TIR)-domain, suggested that NPT1220-312 may be acting by an allosteric non-competitive mechanism. To test this idea, a series of inhibition curves were generated using different concentrations of TLR2 or TLR9 agonists. Schild regression analysis was then applied to the derived curves. NPT1220-312 concentration dependently suppressed the maximum agonist-evoked response by the TLR2 and TLR9 agonists with a minimal rightward shift of the curves (Figure 3). This pattern of inhibition is consistent with an allosteric non-competitive interaction at both TLR2 and TLR9. Gaddum plot analysis demonstrates that NPT1220-312 is a high-affinity antagonist of TLR2 and TLR9 with K_B_ values of 100 nM for TLR2 and 20 nM for TLR9.

### 3.4. NPT1220-312 Blocks TLR2- and TLR9-Mediated Signaling in Peripheral and Central Immune Cells

**Figure 4:**
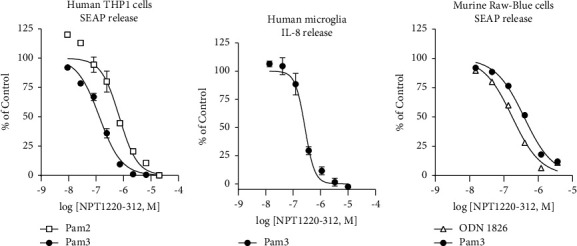
NPT1220-312 blocks TLR2 and TLR9 activation in peripheral and central immune cells. NPT1220-312 blocked: (a) Pam_2_CSK_4_- and Pam_3_CKS_4_-evoked release of SEAP in human THP1 cells, (b) Pam_3_CKS_4_-evoked release of IL-8 in human microglia, and (c) Pam_3_CKS_4_-evoked release of SEAP- and ODN1826-evoked release of SEAP in murine RAW-Blue cells.

NPT1220-312 concentration dependently blocked TLR2-evoked responses in human THP-1 peripheral monocytes, immortalized human microglia, and murine RAW264-Blue macrophage cells. NPT1220-312 blocked Pam_2_CSK_4_ (IC_50_ = 701 nM; 488–1,006 nM) and Pam_3_CSK_4_ (IC_50_ = 132 nM; 113–155 nM) evoked release of SEAP in human THP1 cells and Pam_3_CSK_4_-evoked release of IL8 (IC_50_ = 270 nM; 221–330 nm) in human microglia. NPT1220-312 also blocked Pam_3_CSK_4_-evoked release of SEAP (IC_50_ = 388 nM; 331–454 nM) and ODN1826-evoked release of SEAP (IC_50_ = 175 nM; 147–209 nM) in murine RAW-Blue cells (Figure 4). Thus, NPT1220-312 potently and robustly inhibits cellular inflammatory signaling mediated by both TLR2 and TLR9 in multiple immune cell types with endogenous expression of the receptors.

### 3.5. NPT1220-312 Inhibits Inflammasome Priming/Activation in Differentiated Human Macrophages

**Figure 5:**
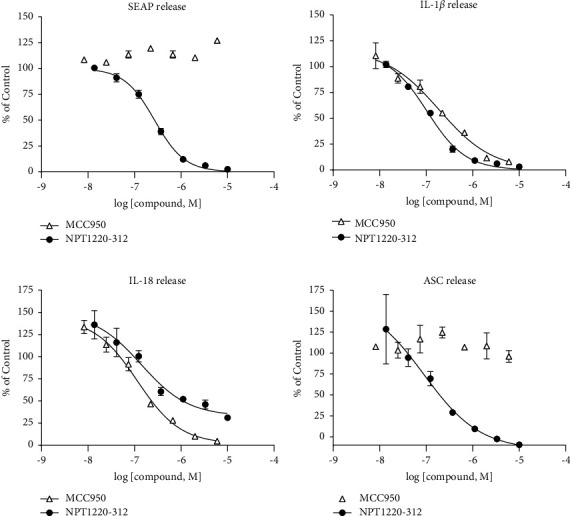
NPT1220-312 blocks TLR2-mediated secretion of inflammasome markers. The NLRP3 inflammasome in THP1 cells differentiated to macrophages was primed with 1.5 ng/ml of Pam_3_CSK_4_ and then activated with 100 *μ*g/ml monosodium urate (MSU): (a) NPT1220-312 but not MCC950 blocked Pam_3_CSK_4_-evoked release of SEAP. (b) Both MCC950 and NPT1220-312 blocked IL-1*β* release. (c) Both MCC950 and NPT1220-312 blocked IL-18 release. (d) NPT1220-312 but not MCC950 blocked Pam_3_CSK_4_-evoked release of ASC.

NPT1220-312 concentration dependently blocked inflammasome signaling that was initiated by priming with Pam_3_CSK_4_ and activated with MSU as measured with IL-18 (IC_50_ = 139 nM; 34–564 nM), IL-1*β* (IC_50_ = 150 nM; 121–163 nM), and ASC (IC_50_ = 215 nM; 111–419 nM). The reference inflammasome inhibitor MCC950 similarly inhibited the release of IL-18 (IC50 = 115 nM; 71–187 nM) and IL-1*β* (IC50 = 319 nM; 220–442 nm) but did not inhibit the production of ASC, nor did MCC950 inhibit Pam_3_CSK_4_-evoked production of SEAP (Figure 5).

### 3.6. NPT1220-312 Blocks TLR2 Signaling in Whole Blood

**Figure 6:**
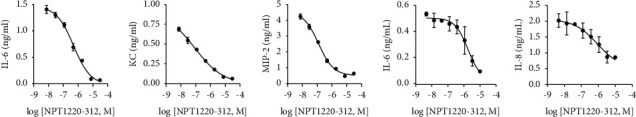
NPT1220-312 blocks Pam_3_CSK_4_-evoked cytokine release in whole blood. NPT1220-312 blocked: (a) Pam_3_CSK_4_-evoked release of: IL-6; (b) murine IL-8 homologues: KC; and (c) MIP-2 in murine whole blood. NPT1220-312 blocked: (d) Pam_3_CSK_4_-evoked release of: IL-6 and (e) IL-8 in human whole blood.

The ability of NPT1220-312 to block TLR2 in situ was evaluated using murine and human whole blood. Activation of TLR2 signaling by Pam_3_CSK_4_ in whole blood induced the release of multiple pro-inflammatory cytokines including interleukin 6 (IL-6), keratinocyte chemoattractant (KC), and macrophage inflammatory protein 2 (MIP-2). NPT1220-312 blocked Pam_3_CSK_4_-induced release of pro-inflammatory cytokines in murine whole blood with potencies similar to those observed in the cell-based assays: IL-6 (IC_50_ = 119 nM; 61–230 nM), KC (IC_50_ = 94 nM; 32.8–271 nM) b), and MIP-2 (IC_50_ = 119 nM; 61–230 nM). NPT1220-312 also blocked Pam_3_CSK_4_-evoked release of IL-6 and IL-8 in human whole blood with IC_50_ values of 1.75 (1.11–2.75) and 0.87 (0.30–0.25) *μ*M, respectively (Figure 6).

## 4. Discussion

In this study, the actions of the novel TL2/TLR9 antagonist NPT1220-312 on TLR-mediated responses in human and murine immune cells and in murine whole blood were characterized. NPT1220-312 was found to be a potent and selective allosteric inhibitor of both TLR2 and TLR9 but to be essentially inactive at other TLRs. NPT1220-312 potently blocked multiple TLR2 and TLR9 agonists in multiple murine and human cell systems. It also blocked the NLRP3 inflammasome-mediated production of IL-18, IL-1*β*, and ASC in human macrophages that were evoked by priming with a TLR2 agonist. Lastly, NPT1220-312 also potently blocked TLR2-mediated release of inflammatory cytokines and chemokines in murine and human whole blood.

Several small-molecule TLR2 and TLR9 inhibitors have been previously described. However, the TLR2 inhibitors that have been thus far disclosed [41–44] are notably weak inhibitors of TLR2-mediated signaling (Table 1). The TLR9 inhibitors that have been described to date include the small molecule E6446 and several oligonucleotide-based compounds. E6446 inhibits both TLR9 and TLR7 and may have a mechanism similar to chloroquine or DNA dyes like Hoechst and propidium iodide [45, 46]. The oligonucleotides that block TLR9 also block TLR7 and/or TLR8. These oligonucleotide antagonists have been tested in mouse models and in humans. However, a major challenge for the use of oligonucleotides in disorders of the CNS is their delivery across the blood-brain barrier. To date, no potent and selective small-molecule dual inhibitors of TLR2 and TLR9 have been described.

NPT1220-312 was discovered through a medicinal chemistry effort that entailed the synthesis of novel chemical entities and their evaluation in reporter cell lines of TLR2 and TLR9. This chemistry effort used structure-based design methods to target the TIR domain of TLR2, a critical site in the transduction of ligand binding to receptor signaling. Targeting the TIR domain, rather than TLR2 agonist recognition sites, provides several potential advantages. First, by targeting the TIR domain, the effective concentration (IC_50_ value) of the antagonist in blocking evoked inflammatory responses is not as markedly diminished by high concentrations of agonist as would be the case for an antagonist that competed for the agonist recognition site. Second, by targeting this domain, it is possible that beneficial phagocytotic actions of TLR2 will be maintained while pro-inflammatory signaling is blocked [18]. And third, by targeting the intracellular TIR domain, these allosteric antagonists effectively block signaling of multiple distinct agonists, even if they may bind to different extracellular recognition sites on the receptor. This is an important consideration since multiple TLR-activating DAMPs may be released from damaged and degenerating neurons, and it may be desirable to block all such activators. The finding that NPT1220-312 also blocked TLR9 was an unanticipated finding that is likely related to structural similarities in the TIR domain of TLR2 and TLR9. Since both TLR2 and TLR9 have been implicated in several neurodegenerative disorders and since both TLR2 and TLR9 blocking agents have been safely administered to humans, dual inhibitors such as NPT1220-312 present a unique therapeutic opportunity to be further pursued.

Numerous studies have found evidence for chronic dysregulation of the immune system in patients with neurodegenerative disorders that is, in part, mediated by TLR2 and TLR9. Both TLR2 and TLR9 protein or mRNA levels are elevated in tissue samples from patients with neurodegenerative diseases including ALS, PD, and GBS [32, 33, 47–49] with patterns of increased inflammatory mediators in peripheral or CSF samples generally corresponding to the inflammatory mediators that are released upon activation of TLR2 and TLR9 (e.g., TNF*α*, IL-18, IL-1*β*, IL-6, IL-8, MCP-1, MIP-1*α*, IL-15, IL-17 IFN-ɣ, and MIP-1*β* in ALS and IL-1*β*, IL-2, IL-4, IL-6, IL-8, TGF-*α*, TGF-*β*, MCP-1, MIP-1*α*, and INF-ɣ in plasma or CSF samples from PD patients). Moreover, in the case of PD, the magnitude and distribution of increased TLR2 receptor expression have been found to be correlated with the duration of the disease and the regional deposition of the disease-associated protein alpha-synuclein [33, 48]. The role of TLR2 and TLR9 in neurodegenerative disorders is further supported by studies with animal models in that both TLR2 and TLR9 protein or RNA has been found to be upregulated in transgenic models of ALS or PD with either genetic or antibody-mediated block of these receptors shown to reduce the expression of inflammatory mediators and associated burden of pathologic proteins [15, 37, 39, 48]. Together, the clinical findings of dysregulated TLR2 and TLR9 signaling and the findings from animal models showing the benefits of blocking TLR2 and TLR9 provide a compelling rationale for developing TLR2/9 antagonists as potential novel therapeutics for neurodegenerative disorders.

One intriguing aspect of the action of NPT1220-312 relates to the NLRP3 inflammasome, a multimolecular complex implicated in a wide range of neurodegenerative and autoimmune disorders. Both TLR2 and TLR9 have been shown to participate in the priming step of the NLRP3 inflammasome, while a wide range of disease-associated pathologic proteins including alpha-synuclein, amyloid-beta, and SOD-1 have been shown to participate in the activation step required for NLRP3 function [19, 21, 22, 26]. In the current study, NPT1220-312 was shown to block the NLRP3-mediated release of IL-18, IL-1*β*, and ASC from human macrophages that was evoked by priming with a TLR2 agonist followed by activation with MSU. This suggests that NPT1220-312 can block a key point in the convergence of neuroinflammation and protein pathology in neurodegenerative disorders. One unanticipated finding from these studies was that in contrast to NPT1220-312, the NLRP3 inhibitor MCC950 [50] did not block the NLRP3-mediated release of ASC. This suggests that MCC950 acts downstream in the signaling cascade divergence of ASC from IL-18 and IL-1*β* while the finding that NPT1220-312 blocked NLRP3-mediated IL-18, IL-1*β*, and ASC production is consistent with an action at the priming step of inflammasome signaling and suggests that targeting this proximal priming step of NLRP3 may be an effective approach to fully abrogating the inappropriate activation of this inflammasome by TLR2 activating DAMPs.

The clinical and preclinical findings pointing to the involvement of TLR2- and TLR9-mediated inflammatory processes in neurodegenerative disorders together with the findings presented here demonstrated that the dual TLR2/TLR9 antagonist NPT1220-312 blocks the release of disease-relevant inflammatory mediators in both peripheral and central immune cell systems and also blocks TLR2-mediated priming of the NLRP3 inflammasome, suggest that compounds such as NPT1220-312 may have therapeutic utility in the treatment of neurodegenerative disorders. One next step in the evaluation of TLR2/9 inhibitors as potential therapeutics is to test whether compounds such as NPT1220-312 have beneficial actions in animal models of neurodegenerative disorders. As a prerequisite to these studies, we tested whether NPT1220-312 blocked TLR2 receptors in situ. The finding that NPT1220-312 blocked the release of multiple cytokines including IL-6, KC, and MIP-2 evoked by TLR2 agonists in murine whole blood supports the use of NPT1220-312 in animal model studies and further provides an estimate to what may be receptor-blocking concentrations of NPT1220-312 in these animals.

## 5. Conclusion

NPT1220-312 is a potent and selective small-molecule TLR2 and TLR9 receptor antagonist that blocks TLR2- and TLR9-mediated inflammatory responses in murine and human cells of either central or peripheral origin as well as in whole murine blood. These findings provide a strong basis for further exploring the potential utility of NPT1220-312 and similar compounds in the treatment of ALS, PD, and other neurodegenerative disorders.

Values are the mean of at least three separate determinations using hTLR2 HEK cells as activated with Pam_2_CSK_4_ or Pam_3_CSK_4_.

## Figures and Tables

**Table 1:** Potencies of NPT1220-312 and other reference inhibitors of TLR2.

Inhibitor	IC_50_ (PAM-2; μM)	IC_50_ (PAM-3; μM)
NPT1220-312	0.7	0.5
C29	57.6	44.4
O-vanillin	61.6	40.2
CU-CPT22	38.9	28.6

## Data Availability

Final study reports with original data are archived at Neuropore Therapies.
